# Wildlife surveillance using deep learning methods

**DOI:** 10.1002/ece3.5410

**Published:** 2019-08-17

**Authors:** Ruilong Chen, Ruth Little, Lyudmila Mihaylova, Richard Delahay, Ruth Cox

**Affiliations:** ^1^ Department of Automatic Control and Systems Engineering University of Sheffield Sheffield UK; ^2^ Department of Geography University of Sheffield Sheffield UK; ^3^ National Wildlife Management Centre Animal and Plant Health Agency Gloucestershire UK

**Keywords:** automatic image recognition, bovine tuberculosis, convolutional neural networks, deep learning, wildlife monitoring

## Abstract

1. Wildlife conservation and the management of human–wildlife conflicts require cost‐effective methods of monitoring wild animal behavior. Still and video camera surveillance can generate enormous quantities of data, which is laborious and expensive to screen for the species of interest. In the present study, we describe a state‐of‐the‐art, deep learning approach for automatically identifying and isolating species‐specific activity from still images and video data.
2. We used a dataset consisting of 8,368 images of wild and domestic animals in farm buildings, and we developed an approach firstly to distinguish badgers from other species (binary classification) and secondly to distinguish each of six animal species (multiclassification). We focused on binary classification of badgers first because such a tool would be relevant to efforts to manage *Mycobacterium bovis* (the cause of bovine tuberculosis) transmission between badgers and cattle.
3. We used two deep learning frameworks for automatic image recognition. They achieved high accuracies, in the order of 98.05% for binary classification and 90.32% for multiclassification. Based on the deep learning framework, a detection process was also developed for identifying animals of interest in video footage, which to our knowledge is the first application for this purpose.
4. The algorithms developed here have wide applications in wildlife monitoring where large quantities of visual data require screening for certain species.

## 1 INTRODUCTION

The use of remote still and video surveillance cameras in wildlife research and management has grown rapidly in recent years (Nguyen et al., 2017; Villa, Salazar, & Vargas, 2017; Zeppelzauer, 2013). The purpose of surveillance may vary widely from identification of pest species or problem behavior to estimating the abundance and distribution of species of conservation importance, but they usually share a common need, which is to identify particular target species. This surge of interest in remote surveillance has, however, been accompanied by increasing recognition of the challenges associated with screening the enormous quantities of image data for the species of interest. The conventional approach of sifting through images by eye can be laborious and expensive (although some studies have reduced costs by crowd sourcing; e.g., Hsing et al. (2018)). Thus, there is considerable interest in the development of automated methods (Zeppelzauer, 2013).

In recent years, machine learning methods for automated recognition of animals have increasingly been used in biological and fisheries monitoring. These technologies have improved the ability to capture high‐resolution images in challenging environments and have consequently led to more effective management of natural resources (Spampinato et al., 2015). However, the method used to detect animals is somewhat specific to the situation. For example, automated detection and tracking of elephants using color to separate the animal from the background have been successful (Zeppelzauer, 2013), and while the approach could be adapted to other species, it would not be applicable where color is absent, for example, for nocturnal species. Animal facial recognition has also been successfully employed for wildlife detection (Burghardt & Ćalić, 2006), although it is clearly only applicable where the face is visible. In addition to automatic recognition from still photographs, recognition by automatic video processing has also been trialed. For example, the dairy sector has used this approach to locate and track dairy cows (Martinez‐Ortiz, Everson, & Mottram, 2013), although one of the challenges here is to be able to distinguish specific individual animals, while rejecting images that contain people or other animals.

Stills cameras and CCTV have been used for many years to monitor wildlife visits to farms in the UK as part of the management of bovine tuberculosis (bTB; e.g., Payne, Chappa, Hars, Dufour, & Gilot‐Fromont, 2016; Robertson et al., 2016, 2017). This disease is a pressing animal health problem in the UK (Defra, 2014), and dealing with bTB in cattle costs the taxpayer an estimated £100 million a year (Defra, 2018). Although cattle often acquire bTB from one another (Donnelly & Nouvellet, 2013), European badgers (*Meles meles*) are a potential source of infection (Murhead & Burns, 1974) and their presence on cattle pasture and in farm buildings provides opportunities for transmission through direct or indirect contact (Drewe, O'Connor, Weber, McDonald, & Delahay, 2013; Garnett, Delahay, & Roper, 2002; Judge, McDonald, Walker, & Delahay, 2011; Tolhurst, Delahay, Walker, Ward, & Roper, 2009; Ward, Tolhurst, & Delahay, 2006). Despite much research, there remains a paucity of evidence on where and when transmission occurs (Godfray et al., 2013), and hence, monitoring of badger behavior in farm environments remains a research priority.

Attempts to monitor badger behavior can be particularly challenging because images are often collected under poor illumination, without color, in changeable weather and from cameras situated at different positions with respect to the monitored area. While CCTV technology can potentially record detailed behavioral data (Tolhurst et al., 2009), it requires regular (often daily) visits to replace batteries or memory cards. As a consequence, most badger surveillance studies have employed stills cameras (e.g., Defra, 2014) as they can remain in the field for several weeks at a time. Despite being motion‐triggered, both approaches produce a large amount of visual data that need to be manually reviewed for target and nontarget species.

To address these challenges, we piloted the use of machine learning methods for automatic recognition of wildlife. In order to classify images, image features are required. Hand‐crafted image feature methods such as the histogram of oriented gradient (HOG; Dalal & Triggs, 2005) and scale‐invariant feature transform (SIFT) have been widely applied (Zhu, Yuen, Mihaylova, & Leung, 2017). However, state‐of‐the‐art automatically learned features by convolutional neural networks (CNNs) have outperformed all the hand‐crafted feature methods on large datasets (Krizhevsky, Sutskever, & Hinton, 2012). Convolutional neural networks have only recently been applied to automatic classification of wildlife images, with limitations in performance reported. For example, Chen, Han, He, Kays, and Forrester (2014) first demonstrated the technique, although their framework was only 38% accurate. Since this time, improvements have been made by training on very large datasets. For example, Gomez, Salazar, and Vargas (2016) developed a CNN to identify wild animals from the world's largest camera trap project published to date, known as the Snapshot Serengeti dataset (3.2 million images of 48 species; Swanson et al., 2015). Overall accuracy for animal identification was not reported, but was estimated at approximately 57% elsewhere (Norouzzadeh et al., 2018). Very recently, Norouzzadeh et al. (2018) applied different CNN architectures including AlexNet (Krizhevsky et al., 2012), VGG (Simonyan & Zisserman, 2015), and ResNet (He, Zhang, Ren, & Sun, 2016) to the same dataset and achieved an accuracy of 92% for species identification. While these methods show improved accuracy, we are not aware of any studies that have considered how to detect wildlife images of interest from film sequences. A specific challenge here is identifying when an animal of interest enters the area in front of the camera. Detecting such images of interest would enable collection of detailed film footage, while optimizing storage space by only saving frames of interest. Here, we aim to develop a robust framework to classify wildlife images, and we then apply the same image recognition algorithm to video footage.

## 2 AIMS

1. Develop an automated image classification algorithm which can identify still images containing badgers, while rejecting those containing other animals.
2. Test, refine, and calibrate the image classification algorithm to identify and classify six different animal species from still photographs.
3. Test, refine, and calibrate the image classification algorithm so that it can be used to identify badger presence in a sample of video footage.

## 3 METHODS

### 3.1 Deep learning for wildlife species recognition

**Figure 1:**
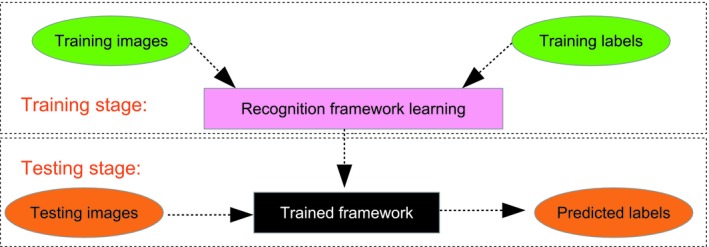
The training and testing processes of a recognition framework

Building an image recognition framework involves a training stage and a testing stage (Figure 1). During the training stage, parameters in the recognition framework are learned from the training images, which have already been labeled by hand (a label being the animal that is shown in the image). During the testing stage, the trained framework takes incoming images as input and outputs a label prediction.

**Figure 2:**
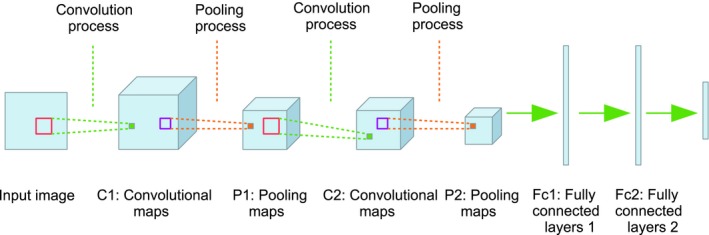
An example of a generic CNN architecture (Chen et al., 2018)

Traditional image recognition frameworks involve separate processes for feature extraction and classification. However, CNNs automatically learn the image features and build a classifier. In this sense, CNNs could be regarded as a “black box” which automatically builds a mapping relationship between the input image and its output label. Inside the “black box,” there are different layers similar to neural networks, where each element in a layer is regarded as a neuron, and each neuron in the current layer is fully connected to neurons in the next layer (Schmidhuber, 2015). Data are transferred from the current layer to the next layer, and the last layer is directly connected to the output label. A typical CNN architecture is mainly composed of convolutional layers (C), pooling layers (P), and fully connected layers (Fc; Chen, Jalal, Mihaylova, & Moore, 2018; Figure 2).

A convolutional layer is composed of different convolutional maps. In a convolution stage, feature maps are convoluted with different kernels, which are equivalent to filters in the field of image processing (Chen et al., 2018). A pooling layer is composed of many different pooling maps. A pooling process is often applied on convolutional layers. A pooling process decreases the size of the input feature maps, which can be regarded as a downsampling stage. As shown in Figure 2, these two processes are repeated. In this figure, a convolutional process is always followed by a pooling operation, although this is not necessary and different CNN structures are valid.

In the current study, we describe the development and testing of two CNN frameworks. The first is a self‐trained framework (CNN‐1) based on a newly created wildlife dataset. The second is a transferred framework based on AlexNet (CNN‐2), which is then fine‐tuned on our wildlife dataset. AlexNet is another CNN‐based model which was trained on one of the world's largest public image datasets known as ImageNet, consisting of 1.2 million labeled images with 1,000 categories (Deng et al., 2009).

Studies have shown that CNNs learned from a large‐scale dataset in the source domain can be effectively transferred to a new target domain (Donahue et al., 2014; Yosinski, Clune, Bengio, & Lipson, 2014). In this transfer learning process, the already trained weights are used as the initial weights and are then fine‐tuned using the task dataset. The assumption is that the network has already learned useful features and could therefore attain greater accuracy than a model trained on a smaller dataset (Nguyen et al., 2017).

We designed two frameworks because each has advantages and disadvantages. A CNN built using a smaller training dataset (CNN‐1) would require less computing memory than one trained on a large dataset (CNN‐2); however, it would be more likely to suffer from overfitting. The performance of a CNN initialized with well‐trained weights from a large dataset (CNN‐2) would be highly dependent on the image similarity between the source domain (ImageNet) and target domain (Wildlife). Given that the two datasets that we used were similar, we expect CNN‐2 to outperform CNN‐1.

#### 3.1.1 CNN‐1

**Figure 3:**
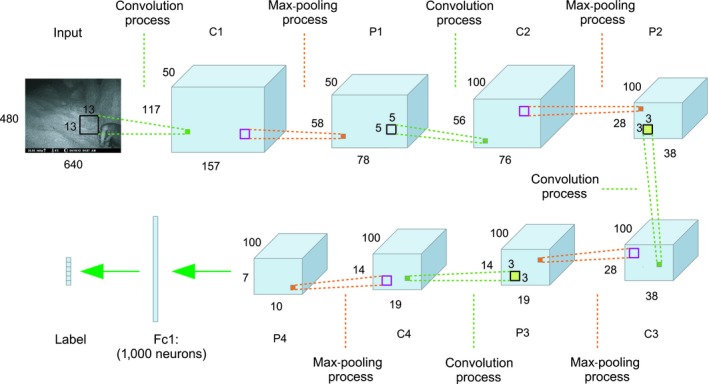
The architecture of CNN‐1

In both CNN‐1 and CNN‐2, the training process aimed to teach the weights in the “black box.” In CNN‐1, all the weights were randomly initialized and updated based on the training data. The CNN‐1 framework consists of four convolutional layers, four max‐pooling layers, and a fully connected layer (Figure 3). The input image of size [480 pixels × 640 pixels × 3 channels] was transferred to 50 convolutional maps of size [117 pixels × 157 pixels] in the first convolutional layer (C1). In the first pooling layer (P1), 50 pooling maps were generated based on C1. This transformation was achieved by using 50 convolutional kernels of size [13 pixels × 13 pixels] with a stride of [4 pixels × 4 pixels]. A stride represents how much the convolution kernels shift during each step on the input. Thus, the convolutional kernels shifted 4 pixels, either along the horizontal axis or along the vertical axis in each step.

The second convolutional process was applied on P1 by using 100 convolutional kernels; hence, 100 convolutional maps were generated in C2. The same process was repeated in P2, C3, P3, C4, and P4. In P4, there were 100 pooling maps of size [7 pixels × 10 pixels]. Elements in P4 were reshaped to a vector form of 7,000 neurons, and these neurons were fully connected to 1,000 neurons in the first fully connected layer (Fc1). Fc1 was then fully connected with the output neurons, which represent the corresponding label information. Appendix 1 details the architecture of CNN‐1.

#### 3.1.2 CNN‐2

**Figure 4:**
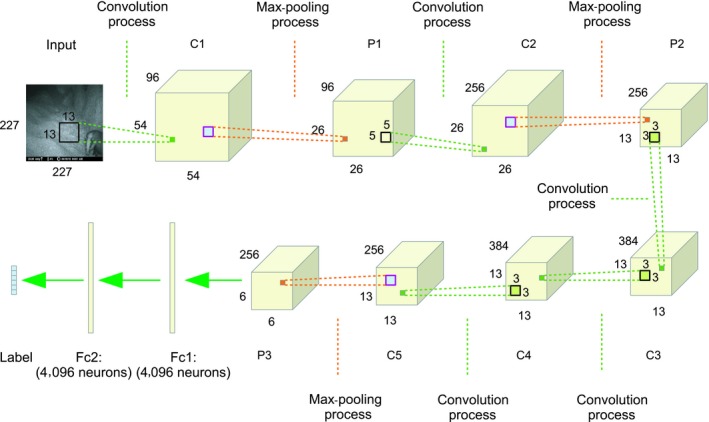
The architecture of CNN‐2

The weights of CNN‐2 were learned from the trained model AlexNet. CNN‐2 kept all the weights except for the last three layers from AlexNet. The output layer was self‐defined, and weights were fine‐tuned based on the badger dataset. The developed CNN‐2 framework (Figure 4) has five convolutional layers, three max‐pooling layers, and two fully connected layers. Appendix 2 details the architecture of CNN‐2.

### 3.2 Trained CNNS applied to video footage

The trained CNNs were directly applied to video footage, because film can be considered as a sequence of image frames. In order to speed up the detection process, all images were converted to grayscale. If an image was detected as a potential frame of interest, then the color framework was used for recognition. Images of interest were those that contained objects of interest (any animal). In film footage, the movement of an animal results in pixel value variations in adjacent frames. Intuitively, differences between adjacent frames could therefore be calculated. Here, instead of directly applying frame differences, a dynamic background (B) was used with the following updating process:

(1) $$B_{\mathrm{ij}}^{t}=\left(1-\alpha \right)\times I_{\mathrm{ij}}^{t}+\alpha \times B_{\mathrm{ij}}^{t-1}$$

where *i*; *j* represents the vertical and horizontal pixel location, I is the input frame, and $I_{\mathrm{ij}}^{t}$ represents the current pixel value at the location index (*i, j*). The initial B is set as the first input frame and dynamically updates, hence, the difference between the current frame and the background is given by:

(2) $$D_{\mathrm{ij}}^{t}=\left|I_{\mathrm{ij}}^{t}-B_{\mathrm{ij}}^{t}\right|$$

where |·| calculates the absolute value.

However, frame difference does not necessarily indicate that there is an animal present, since differences can also be caused, for example, by moving vegetation. Here, the following assumptions were made in order to decrease the false‐positive detection rate: (a) if an animal moves, the frame difference should be relatively large and (b) the movement of the animal is the main cause of the pixel changes, and the camera is not occluded by the animal's body.

In order to remove tiny variations, a dynamic threshold process was applied based on the maximum value among all *D^t^_ij_*:

(3) $$D_{\mathrm{ij}}^{t}=\left\{\begin{array}{c} 0\;\mathrm{if}D_{\mathrm{ij}}^{t}<\beta \cdot \text{max}\left(D^{t}\right),\\ D_{\mathrm{ij}}^{t}Otherwise \end{array}\right.$$

This process was followed by a median filter aimed at removing noise. Animal movements tend to happen in a small area; therefore, if a large area is moving, it is likely that the camera is either moving or it has been blocked by an animal's body. Hence, a frame was omitted when its D had nonzero values that were either too small or too large. Here, animal size was restricted to 200 pixels and half of the total pixels of the image. Frames with large pixel variations were removed in order to decrease the false‐positive detection rate caused by other factors such as camera movement, windy weather, and a suddenly changing scene.

For the considered frames, an energy term *E^t^* can be calculated by summing all the nonzero values in *D^t^*:

(4) $$E^{t}=\Sigma_{ij}D_{\mathrm{ij}}^{t}$$

The average variation of each pixel is given by:

(5) $$y^{t}=E^{t}/n^{t}$$

where *n* is the number of nonzero value pixels in *D*. For frames with animal motion, the image should have large total energy *E^t^*. In addition, the pixel variation made by animals should be larger than other factors; thus, the variation made by animal objects should be the main portion of the total energy, and therefore, its *y* should be large. Hence, by comparing the *y^t^* with a threshold, the *t*th frame would be sent to the classification stage if its *y* value was beyond the threshold.

**Figure 5:**
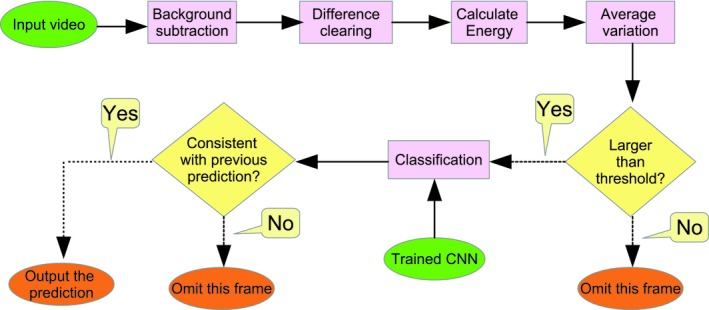
The process of applying trained CNNs to video footage

If an animal is detected, the classification result should be consistent within a short period of time (e.g., 0.1 s). Therefore, a confirmation process (as shown in Figure 5) was applied in order to decrease the number of false positives. When the prediction agreed with the previous prediction, the classification result was confirmed as the output.

### 3.3 Processing

The performance evaluations of CNN‐1 and CNN‐2 were conducted in MATLAB on a desktop PC with the following specification: Intel I7‐7700K (4.2 GHz × 4), 16 GB of RAM, and an Nvidia GeForce RTX 2080. In the training stage, the choice of optimizer was stochastic gradient descent with momentum of 0.9, and batch sizes were set to 128. We trained CNN‐1 for 200 epochs with an initial learning rate of 0.001 since weights were randomly initialized. Since CNN‐2 was pretrained on another dataset, we trained it for 50 epochs using an initial learning rate of 0.0001. In CNN‐2, images were reshaped to [227 × 227] in order to transfer the weights, while in CNN‐1 images were reshaped to [480 × 640].

### 3.4 Dataset generation

**Figure 6:**
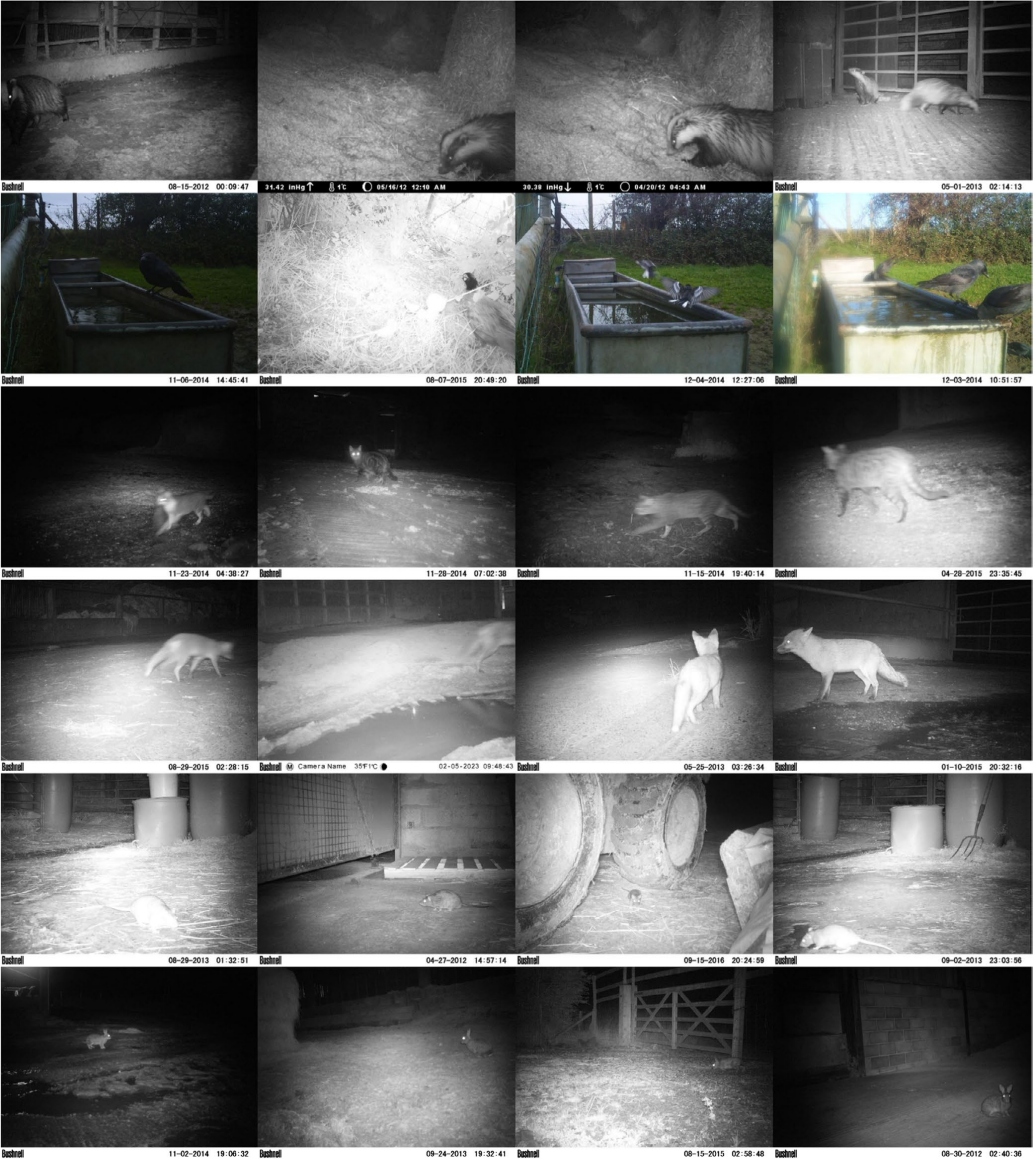
Example images from the testing dataset. From the first row to the last row are badger, bird, cat, fox, rat, and rabbit

The photograph images were captured at a selection of UK farms where surveillance had taken place. All were manually assigned to either badger, bird, cat, fox, rat, or rabbit (Figure 6). We randomly selected 70% of the images to be used in the training process, and the rest were assigned for testing (Table 1). Images were selected randomly in order to provide a diverse variety for training and testing. Training and testing images could not be separated by farm because some farms generated a large proportion of images and because certain animals tended to occur only on one farm.

**Table 1:** Number of images per category in the wildlife dataset

Category	Total images	Training images	Testing images
Badger	1,556	1,089	467
Bird	1,528	1,070	458
Cat	1,083	758	325
Fox	2,693	1,885	808
Rat	570	399	171
Rabbit	938	657	281
Total	8,368	5,858	2,510

To design and evaluate automatic classification, two different scenarios were considered: (a) binary classification distinguishing an image as either belonging to the badger or the nonbadger category and (b) multiclassification to identify an image as one of six animal species.

## 4 RESULTS

### 4.1 Badger versus nonbadger classification

CNN‐1 and CNN‐2 were evaluated for their binary classification performance by apportioning results to four categories: True positives (TP) were the number of badger test images that were correctly classified as badgers, and false positives (FP) were the number of nonbadger test images that were wrongly classified as badgers. False negatives (FN) were the number of badger test images which were wrongly classified as being in the nonbadger category, and true negatives (TN) were the number of the nonbadger test images that were correctly classified as belonging to the nonbadger category. Accuracy represents the ratio between correctly classified images and total images. The F1 score is the harmonic average of the precision (TP/(TP + FP)) and recall (TP/ (TP + FN)) with values from 0 to 1.

(6) $$\text{Accuracy}=\frac{\text{TP}+\text{TN}}{\text{TP}+\text{FP}+\text{FN}+\text{TN}}$$

(7) $$\text{F1 score}=\frac{2\text{TP}}{2\text{TP}+\text{FP}+\text{FN}}$$

#### 4.1.1 Performance of CNN‐1

The CNN‐1 framework had an accuracy of 95.58% (Table 2). The false‐negative rate (17.77%) was much higher than the false‐positive rate (1.37%). This is because there were unbalanced data in each category, which resulted in a test image having a higher probability of being allocated to the majority group in the training dataset. In order to decrease this effect, a resampling process was applied to the minority group. Specifically, images in the badger category were resampled four additional times in order to provide an equivalent number of images in both categories. This resampling process dropped the false‐negative rate from 17.77% to 10.71% and improved the F1 score from 0.87 to 0.89.

**Table 2:** The performance of CNN‐1 for binary classification without and with the resampling process

	Test data			
	Badger	Nonbadger	Accuracy (%)	F1 score
Without resampling				
Prediction				
Badger	384 (TP)	28 (FP)	95.58	0.87
Nonbadger	83 (FN)	2015 (TN)		
With resampling				
Prediction				
Badger	416 (TP)	53 (FP)	95.86	0.89
Nonbadger	51 (FN)	1990 (TN)		

#### 4.1.2 Performance of CNN‐2

CNN‐2 performed better than the CNN‐1 framework (Table 3). Since the unbalanced training dataset caused biased results (described above), we assessed CNN performance using the training dataset both with and without a resampling process.

**Table 3:** The performance of CNN‐2 for binary classification without and with the resampling process

	Test data			
	Badger	Nonbadger	Accuracy (%)	F1 score
Without resampling				
Prediction				
Badger	429 (TP)	22 (FP)	97.61	0.93
Nonbadger	38 (FN)	2021 (TN)		
With resampling				
Prediction				
Badger	442 (TP)	24 (FP)	98.05	0.95
Nonbadger	25 (FN)	2019 (TN)		

The greatest accuracy was achieved using CNN‐2 with a value of 97.61%, increasing to 98.05% with resampling.

### 4.2 Multiclassification

#### 4.2.1 The performance of CNN‐1

For multiclassification, the F1 score is not valid, and instead, the accuracy and mean accuracy were used to evaluate performance. Mean accuracy was obtained by averaging the accuracies from individual categories. We use mean accuracy because it provides a less biased measurement than accuracy when the dataset is not balanced. In the training stage, when using an unbalanced dataset, the weights may be biased toward larger groups, and so, a random test image is more likely to be allocated to a larger group. For example, given 100 testing images which contain 95 badger images and one image in another category, the general accuracy would be 95% if all images were classified to the badger category, while the mean accuracy would be 17.67% (the accuracy in the other animal categories would be zero).

The accuracy of CNN‐1 was 83.07% and the mean accuracy was 79.98%, both of which were lower than for the binary classification (Table 4). As above, a resampling process was applied to the training dataset. During this process, the fox category, which contained the most images, was not resampled, while the others were resampled so that the number of images was similar to the fox category. Thus, the badger and bird categories were resampled once, cat and rabbit twice, and rat four times. The resampling process improved the accuracy of categories that had less training data, such as cat, rat, and rabbit. Overall, the accuracy of CNN‐1 improved slightly to 83.51% and 82.71%, respectively, with resampling (Table 5).

**Table 4:** The performance of CNN‐1 for multiclassification without the resampling process

	Test data						Accuracy (%)	Mean accuracy (%)
	Badger	Bird	Cat	Fox	Rat	Rabbit		
Prediction								
Badger	395	2	6	29	9	6	83.07	79.98
Bird	1	441	3	7	2	4		
Cat	4	3	207	34	10	6		
Fox	54	11	90	704	24	46		
Rat	7	0	5	3	122	3		
Rabbit	6	1	14	31	4	214		
Individual accuracy (%)	84.58	96.29	63.69	87.13	71.35	76.87		

**Table 5:** The performance of the CNN‐1 for multiclassification with the resampling process

	Test data						Accuracy (%)	Mean accuracy (%)
	Badger	Bird	Cat	Fox	Rat	Rabbit		
Prediction								
Badger	402	2	2	34	8	5	83.51	82.71
Bird	0	438	4	12	0	3		
Cat	4	3	235	41	8	4		
Fox	11	0	9	652	12	29		
Rat	11	0	9	7	132	3		
Rabbit	5	5	14	62	11	237		
Individual accuracy (%)	86.08	95.63	72.31	80.69	77.19	84.34		

#### 4.2.2 Performance of the CNN‐2

CNN‐2 had an accuracy of 90.32% for multiclassification (Table 6). Accuracies were higher and more balanced than results achieved using CNN‐1. The lowest accuracy (77.23%) was in the cat category, which can be explained by their resemblance to other animal images, in particular foxes, especially when viewed from behind. The resampling process did not have a considerable influence on the performance of CNN‐2 (Table 7) because the well‐trained weight from AlexNet was less likely to suffer from overfitting. Such overfitting means the model performs perfectly on the training dataset, but does not perform well on the testing dataset.

**Table 6:** The performance of CNN‐2 for multiclassification without the resampling process

	Test data						Accuracy (%)	Mean accuracy (%)
	Badger	Bird	Cat	Fox	Rat	Rabbit		
Prediction								
Badger	431	1	6	10	3	11	90.32	87.57
Bird	2	447	3	5	3	5		
Cat	3	0	251	8	6	5		
Fox	16	5	47	763	10	15		
Rat	5	2	3	6	133	3		
Rabbit	10	3	15	16	16	242		
Individual accuracy (%)	92.29	97.60	77.23	94.43	77.78	86.12		

**Table 7:** The performance of CNN‐2 for multiclassification with the resampling process

	Test data						Accuracy (%)	Mean accuracy (%)
	Badger	Bird	Cat	Fox	Rat	Rabbit		
Prediction								
Badger	434	3	6	24	2	11	86.85	87.04
Bird	2	439	1	2	3	4		
Cat	13	2	281	90	7	7		
Fox	7	1	26	644	7	8		
Rat	6	5	2	13	137	6		
Rabbit	5	8	9	35	15	245		
Individual accuracy (%)	92.93	95.85	86.46	79.70	80.12	87.19		

### 4.3 Detection and classification in video footage

**Figure 7:**
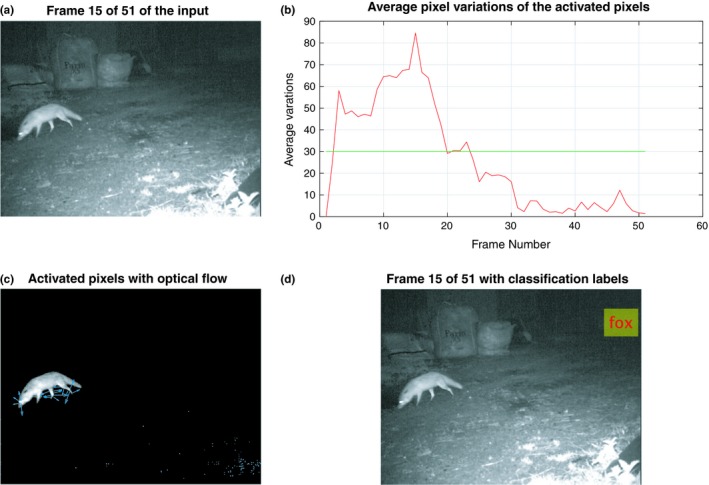
An example of a detected active frame. (a) An input frame; (b) the average variation of the activated pixels in the current frame, which is calculated in Equation 5; (c) the activated pixels in Equation 4, with the blue arrow indicating the estimated movement in the next frame; (d) the classification result of the frame of interest

We applied the trained CNN‐2 to video footage classification (Figure 7). During this process, adjacent frames are compared to determine the mean variation of pixel values (integers from 0 to 255) within an area. A threshold was applied so that any frame with an average variation above this value was sent for classification. The threshold value influences the false‐positive and false‐negative rate. For example, a high threshold would only detect animals when large movements occur, while a lower threshold would result in small background movements being mistakenly identified as animals. Since we included a checking process between adjacent frames, we applied a relatively low threshold value (20), so that only active frames that are highly likely to contain animals are sent for classification. Our analysis demonstrates that CNN‐2 is able to detect movement in adjacent frames and could identify badger presence in a sample of video footage.

## 5 DISCUSSION

Two deep learning frameworks were developed to automatically recognize animal images. We demonstrated high levels of performance for both frameworks, achieving accuracy of 95.86% and 98.05%, for binary classification. For multiclassification, they achieved accuracies of 83.07% and 90.32%, respectively. Our results indicated that the models are robust despite unbalanced data and that they improved with resampling.

While our results are not directly comparable with other frameworks because different datasets have been used, it is relevant to discuss the accuracy of other frameworks if they are to be used for practical purposes. The accuracy of our framework for binary classification was greater than that recorded in other recent work. Nguyen et al. (2017), for example, achieved accuracy of 91.5%–96.6% when detecting images containing wild animals.

For multiclassification, the accuracy of our CNN was comparable to that of another recent CNN, which reported 89.16%–90.4% accuracy for three species; however, our CNN outperformed this network, (84.39% accuracy) when classifying among six species (Nguyen et al., 2017). Our results for multiclassification yielded only slightly lower accuracy than the 93.8% recorded by Norouzzadeh et al. (2018), which used the Snapshot Serengeti database of more than 3 million images for training purposes.

In the present study, CNN‐2 was the more accurate classification framework. An additional advantage was that, since the weights for CNN‐2 were already pretrained, the training time of 2,289 s was considerably less than the 6,076 s required for CNN‐1. Subsequently, the weights only needed to be fine‐tuned to our wildlife monitoring application by using our dataset. Fine‐tuning was advantageous, since we determined that using AlexNet without the pretrained weights resulted in accuracy dropping to 89.68% and 70.28%, respectively, for binary and multiclassification, respectively. Our use of transfer learning is a critical departure from other wildlife recognition frameworks which have trained all the weights using the target datasets (Nguyen et al., 2017; Norouzzadeh et al., 2018; Villa et al., 2017). One drawback of our more accurate framework is that it required more computational memory, 200 Mb for CNN‐2 compared to 20 Mb for CNN‐1. In addition, the implementation stage also took longer to complete. Specifically, CNN‐2 took 18 s to recognize all of the testing images (2,510), while CNN‐1 took only 8 s.

Both deep learning frameworks presented here are data‐driven, and an unbalanced dataset is likely to be more biased toward groups that have more training images. Our resampling process allowed us to understand how an equally balanced training dataset might influence the accuracy of the CNNs. The results show that resampling decreased the error bias in both binary and multiclassification, but was not necessary to improve the accuracy. For example, during binary classification by CNN‐1, the number of false negatives changed from 83 to 51 and the number of false positives changed from 28 to 53 after resampling, while the accuracy did not change considerably (95.58% and 95.86%, respectively). During multiclassification, the accuracy of CNN‐2 for the categories containing more data (badger, bird, and fox) was greater without resampling than with resampling, while accuracy for categories with less data (cat, rat and rabbit) was greater with resampling. Note that a resampling process does not necessarily improve the general accuracy, although it does decrease the variance.

As well as using the recognition framework for binary and multiclassification, in the present study we demonstrated its utility in identifying and isolating badger activity in film footage. For such footage, the recognition results for adjacent frames must be consistent; this checking process decreased the probability of misrecognition. For example, the probability of a cat image being classified as a fox is 14.46%. However, the framework only displayed an incorrect result if two adjacent cat images were both misclassified as foxes. The probability of this occurring was very low (2.09% (0:142)) if these two images are considered to be independent (which they may or may not be).

The ability to identify and isolate badger activity from surveillance footage has multiple benefits. In the short term, it would enable more efficient and cost‐effective analysis of existing footage, and in the longer term, it could allow such surveillance to be extended to more farms and more locations within farms. Ultimately, this work could inform new approaches to managing TB spread between cattle and wildlife (e.g., improved biosecurity to limit opportunities for disease transmission) and could potentially help address some of the social factors that influence disease management at a farm level. For instance, farmers tend to underestimate the level and frequency of badger visits to their farm holdings, suggesting a lack of awareness of the need to prevent badger access to buildings and feed stores (Robertson et al., 2017). Furthermore, research on improving biosecurity (e.g., limiting cattle–badger interactions) indicates that farmers require evidence on the efficacy of prevention measures (e.g., raising cattle troughs, installing badger exclusion measures on feed stores) before they will implement them, and yet little evidence is available (Enticott, Franklin, & Winden, 2012; Gunn, Heffernan, Hall, McLeod, & Hovi, 2008; Little, 2019). Remote monitoring facilitated by automatic recognition analysis could help to address this knowledge gap. Further applications could include the development of a system, whereby real‐time (or near real‐time) alerts could be generated when certain images are identified. This could allow farmers to react to contemporary badger activity on their farm and help them to identify areas for improvement (e.g., to prevent badger entry or badger–cattle contact).

On a wider scale, our work may have applications in many other areas of wildlife management and conservation. We demonstrated rapid classification of thousands of images. Specifically, manual image classification took a minimum of 2 s per image or at least 84 min to classify 2,510 images. In contrast, classification by CNN‐2 saved considerable time, taking only 8 s to classify 2,510 images (0.003 s per image). The ability to process large volumes of photograph and film images, perhaps in real time, allows the possibility for more detailed or larger‐scale studies. It could, for example, facilitate the capture of more definitive evidence that animals visiting vaccine bait stations are actually obtaining baits (Bjorklund et al., 2017; Robertson et al., 2015). It could also make monitoring of wildlife use of road tunnels (e.g., Defra, 2015; PTES, 2018) more feasible or allow the presence of a species of interest to be confirmed, while discarding footage of other species using the same location. It could also allow analysis of existing, underexploited datasets. For example, the National Wildlife Management Centre holds a dataset of more than 100,000 hr of film from farm surveillance, which at the present time cannot be analyzed owing to limited resources.

One additional output of this work is a new image dataset, which contains 8,368 images belonging to six categories: badger, bird, cat, fox, rat, and rabbit. This is an important resource, because prior to the Snapshot Serengeti dataset being made available in 2015 (Swanson et al., 2015), there was no publicly available dataset that the computer science community could use to develop an automated framework for camera trap images. Our dataset is therefore a valuable resource for the transfer learning process of any automatic wildlife framework project.

Currently, our recognition framework is unable to recognize more than one animal category in the same image, nor can it recognize how many animals are present. Adaptations to enable these features would allow automatic estimation of ecologically important metrics such as population abundance and diversity. Indeed, recent work on two classifiers has shown promise in quantifying animal species with accuracies of between 77% and 93% (Schneider, Taylor, & Kremer, 2018). Further work is also required to develop this approach to make it more accessible to wildlife researchers. A nonexpert can run the software developed here by using only the executable version of the code. However, the development of an interactive interface menu is required for a more user‐friendly tool. Our work has proven the feasibility of automating species‐specific recognition, but the bespoke application of this technology, in the form of a program or web‐based service, requires further development.

In summary, we focused on three tasks where very little work has been conducted in a rapidly growing field of research, namely using CNN (a) for automatic wild animal detection, (b) to filter out nonanimal images, and (c) for wild animal recognition from film footage. Our approach to automated wildlife recognition can overcome a major obstacle in camera trap surveillance. The ability to collect data automatically, at little cost and with a high level of accuracy, could have a significant positive impact on wildlife research and management.

## CONFLICT OF INTEREST

None declared.

## AUTHOR CONTRIBUTIONS

All authors contributed to conceiving the ideas and designing the methodology. RC and RD provided the photographic data. RChen and LM developed the CNNs and analyzed the data. RChen and RC interpreted output and led the writing of the manuscript. All authors contributed critically to the drafts and gave final approval for publication.

## Data Availability

Photograph dataset available from ORDA: The University of Sheffield Research Data Catalogue and Repository.

## APPENDIX 1 Architecture of CNN‐1

**Table:**

Require
Load images from the source, weights are randomly initialized
Ensure
1. Image input layer [480 × 640 × 3] with “zero center” normalization
2. Convolution layer 1. 50 [13 × 13 × 13] convolution kernels with stride of [4 4], no padding is applied
3. ReLU nonlinear function 1
4. Max pooling 1, with size [3,3] with stride 2, no padding is applied
5. Batch normalization 1
6. Convolution layer 2. 80 [5 × 5 × 50], with padding [1 1 1 1] applied ([top bottom left right])
7. ReLU nonlinear function 2
8. Max pooling 2, with size [2 2] with stride [2 2], no padding is applied
9. Batch normalization 2
10. Convolution layer 3. 100 [3 × 3 × 80] with stride of [1 1], with padding [1 1 1 1] is applied
11. ReLU nonlinear function 3
12. Max pooling 3, with size [2 2] with stride [2 2], no padding is applied
13. Batch normalization 3
14. Convolution layer 4. 100 [3 × 3 × 100] with stride of [1 1], with padding [1 1 1 1] is applied
15. ReLU nonlinear function 4
16. Max pooling 4, with size [2 2] with stride [2 2], with padding [0 0 0 1] is applied
17. Batch normalization 4
18. Fully connected layer 1, with 1,000 neurons
19. ReLU nonlinear function
20. Dropout layer, with probability is set to 0.5
21. Fully connected layer 2, with 6 neurons. (If the task is only to distinguish badger and nonbadger, change the number of neurons to 2.)
22. Softmax layer
23. Classification layer

## APPENDIX 2 Architecture of CNN‐2

**Table:**

Require
Resize the input images to the size of [227 × 227 × 3] (image size [227 × 227] and image channel [3])
Ensure
1. Image input layer [227 × 227 × 3] with “zero center” normalization
2. Convolution layer 1. 96 [11 × 11 × 3] convolution kernels with stride of [4 4], no padding is applied
3. ReLU nonlinear function 1
4. Max pooling 1, with size [3 3] with stride 2, no padding is applied.
5. Batch normalization 1
6. Convolution layer 2. 256 [5 × 5 × 48], with padding [1 1], padding size of [2 2 2 2]
7. ReLU nonlinear function 2
8. Batch normalization 2
9. Max pooling 2
10. Convolution layer 3. 384 [3 × 3 × 256] with stride of [1 1], with padding [1 1 1 1]
11. ReLU nonlinear function 3
12. Convolution layer 4. 384 [3 × 3 × 192] with stride of [1 1], with padding [1 1 1 1]
13. ReLU nonlinear function 4
14. Convolution layer 5. 256 [3 × 3 × 192] with stride of [1 1], with padding [1 1 1 1]
15. ReLU nonlinear function 5
16. Max pooling 5
17. Fully connected layer 1, with 4,096 neurons
18. ReLU nonlinear function
19. Dropout layer 1, with probability set to 0.5
20. Fully connected layer 2, with 4,096 neurons
21. ReLU nonlinear function
22. Dropout layer 2, with probability set to 0.5
23. Fully connected layer 3, with 6 neurons. (If the task is only to distinguish badger and nonbadger, change the number of neurons to 2.)
22. Softmax layer
23. Classification layer
